# Differentiating Men With Sexual Interest in Children and Those Involved in Sexual Behaviour With Children

**DOI:** 10.1177/08862605251403606

**Published:** 2026-03-03

**Authors:** Delanie Woodlock, Tyson Whitten, Michael Salter, Maria Lamond, Carleigh Slater, Deborah Fry

**Affiliations:** 1 University of New South Wales, Sydney, Australia; 2 University of Edinburgh, Scotland, UK

**Keywords:** Offenders, perpetrators, child maltreatment, sexual abuse, sexual assault

## Abstract

This article examines the factors associated with different groups of men based on their self-reported sexual interest and behaviour towards children. Knowledge in this area usually draws on forensic or clinical samples or relies on self-reporting from cohorts who identify as sexually attracted to children but claim to be non-offending. Clear definitions are therefore needed to interpret findings consistently. In this study, sexual interest in children refers to attraction to individuals aged 15 or younger or interest in abusing a child in hypothetical scenarios. Sexual behaviour involving minors refers to self-reported sexual contact involving individuals under the age of 18. Given the variation in age-of-consent laws within and between jurisdictions and over time, not all behaviours described in this study necessarily constitute criminal offending. Using data from an online survey of 4,918 men representative of the Australian, United States, and United Kingdom adult male population, the current study conducted a series of logistic regression analyses based on Least Absolute Shrinkage and Selection Operator variable selection with *k*-fold cross-validation to identify the covariates independently associated with men reporting (a) no sexual interest or behaviour involving minors, (b) sexual interest without behaviour, (c) sexual behaviour without interest, and (d) sexual interest and behaviour. Most respondents (83.5%) reported neither interest nor behaviour, 5.5% reported sexual interest only, 6.4% reported sexual behaviour without interest, and 4.6% reported interest and behaviour. Attitudes towards child sex abuse consistently distinguished each group of men across multivariable models. Generally, men who reported both sexual interest and behaviour were more distinguishable than any other group, particularly concerning watching violent pornography, anxiety, depression, and attitudes minimising the abusiveness of sexualising children. These findings highlight distinct risk profiles in a representative population sample, which could inform public health prevention strategies and support service responses.

## Introduction

In this article, we examine factors that distinguish men based on their self-reported sexual interest and behaviour towards minors. Understanding these differences is essential for evidence-based prevention, intervention, and responses to child sexual abuse. It is often erroneously assumed that all child sex offenders are sexually interested in children and that all people with a sexual interest in children offend against them (Seto, 2018). Increasing attention is being paid towards people with paedophilic or hebephilic interests who are not abusing children (Cantor & McPhail, 2016), while some individuals who offend against children appear to be driven by opportunity or other criminogenic factors rather than specific sexual preferences (Camilleri & Quinsey, 2008). However, it is currently unknown what proportion of child sex offenders evince a sexual interest in children, how many people in the community have such sexual interests but do not act on them, or the characteristics that distinguish these groups. In the absence of this information, it has been difficult for child sexual abuse scholars, clinicians, and policymakers to adjudicate conflicting claims about the relationship between sexual attraction and sexual offending against children, and the respective roles of preference and opportunity in child sexual abuse. This paper begins with an overview of these debates before presenting the findings of an international survey of 4,918 men and the factors that distinguish those reporting (a) neither sexual interest nor behaviour involving minors, (b) sexual interest without behaviour, (c) sexual behaviour without interest, and (d) sexual interest and behaviour. The concept of ‘*sexual interest*’ in children that is used in this study refers to men who indicated sexual attraction to children or endorsed hypothetical scenarios of sexual contact with a child. This construct is both analytic and descriptive, rather than clinical. In this study, the term ‘minor’ refers to any individual under the age of 18, in accordance with international child protection standards. Because ages of consent vary across jurisdictions, this design choice means that a proportion of the behaviours reported would be lawful in some contexts. Accordingly, these estimates describe sexual behaviour involving persons under 18 and should not be interpreted as the prevalence of criminal offending. The study reveals significant demographic, behavioural, and health differences among these groups, highlighting areas of potential public health intervention and service response.

## The Relationship Between Sexual Interest in Children and Child Sexual Offending

Evidence from clinical and research settings shows that child sexual offending does not always stem from a sexual interest in children, and conversely, that many people with such an interest do not go on to offend. Such distinctions have long been recognised in forensic scholarship. For instance, ‘regressed’ offenders have been characterised as individuals who lack a specific sexual interest in children, and whose offending emerges from a combination of factors, including low self-esteem, a lack of coping skills, immaturity, and life stressors (Groth & Birnbaum, 1978; Lanning, 1995). More recently, scholars such as Wortley and Smallbone (2006) have described a cohort of situational offenders who have no strong sexual interest in children. They are characterised by a lack of self-control and offend opportunistically within a pattern of both sexual and non-sexual criminal activity. Osbourne and Christensen (2020) labelled a specific group of offending men with no sexual interest towards children as following a ‘problems pathway’ beset by health problems, drug and alcohol abuse, social isolation and sexual preoccupation. Contemporary scholarship describes some child sex offenders as ‘opportunistic’, without a sexual preference for children but willing to use children for sexual gratification, and others as ‘situational’ offenders who sexually abuse children due to stressors in their lives (Commonwealth of Australia, 2017).

In general, research suggests that men with a sexual interest towards children have higher levels of sexual preoccupation and deviancy compared to men who do not. Certain factors, such as higher levels of hypersexuality and adverse childhood experiences, appear to distinguish between offenders and non-offenders among men with sexual interest towards children (Cohen et al., 2017; Gerwinn et al., 2018; Stephens et al., 2023). Men who have sexual interest and offend against children have been described in typology scholarship as ‘fixated’ or ‘preferential’ offenders, characterised by sexual fantasies towards children as well as other deviant sexual interests (Groth & Birnbaum, 1978; Osbourne & Christensen, 2020). For such offenders, the sexual abuse of children may be their only or primary criminal activity; that is to say, child sex offending is not part of a broader pattern of criminal or antisocial behaviour. Since a superficially prosocial and respectable façade camouflages their offending, some offenders within this group may abuse multiple victims undetected over a long period (Nicol et al., 2022).

Attempts to understand the proportion of child sex offenders who are sexually attracted to children have been limited by methodological complexities. Phallometric testing, for example, suggests that a high number of offenders, particularly those who are multi-victim offenders, have paedophilic preferences (Freund & Watson, 1991). These findings show that 78.2% of multi-victim offenders against girls and 88.6% against boys had a sexual preference for children (Freund & Watson, 1991). However, these studies are often limited by sampling biases, with the participants usually being convicted sex offenders, who may not be representative of most offenders in the community (Green, 2002; Smiljanich & Briere, 1996).

In contrast, research has increasingly focused on those with sexual interest towards children who do not offend. Indeed, research suggests that there are a group of ‘non-offending’ paedophiles who constitute an important demographic for prevention and early intervention (Cantor & McPhail, 2016). However, the size of this population is largely unknown. Some studies of ‘non-offending’ people sexually interested in children have included men with convictions for child sex offences on the basis that they are not currently offending against children (Stevens & Wood, 2019), while attempts to recruit ‘non-offending’ paedophiles have, in some cases, recruited a sample where the majority reported offending behaviour (Beier et al., 2015). Available research suggests that men with sexual interest towards children who have not offended are more likely to be younger, not have children, express higher attraction to girls versus boys, and have lower levels of childhood adversity when compared to men who have sexually offended against children (Cohen et al., 2017; Jahnke et al., 2023; Schaefer et al., 2010). These studies highlight several similarities between offending and non-offending men with sexual interest towards children, which include high levels of psychological distress, similar education levels, attraction to adult men and women, feelings of stigma, lower levels of self-esteem and social confidence, and distancing from friends (Cohen et al., 2017). Gerwinn et al. (2018) found that there was little statistical differentiation between offenders and non-offenders; however, men who were non-offending had lower lifetime substance use disorders, hyperactivity and impulsiveness.

In light of ongoing debates over the relationship between sexual interest and offending against children, and the lack of empirical information about the characteristics that might distinguish between the two, this paper aims to examine the factors uniquely associated with distinct categories of men based on their sexual interest and behaviour towards children. Specifically, using pooled data from an online survey of 4,918 Australian, U.S., and U.K. men, we examined the demographic characteristics, online behaviours, mental health, social support, adverse childhood experiences (ACE), and attitudes towards child sexual abuse that were independently associated with men who have (a) no sexual interest or behaviour towards children, (b) sexual interest without behaviour, (c) sexual behaviour without interest, and (d) sexual interest and behaviour. In this exploratory study, we first present the descriptive statistics and unadjusted associations for each factor. Next, variable selection was conducted via a series of penalised regression (Least Absolute Shrinkage and Selection Operator [LASSO]) models with *k*-fold cross validation (to reduce overfitting and maximise reliability of the results). The selected covariates were then examined in separate logistic regression models to identify the factors independently associated with each outcome category.

## Methods

### Data

An online survey was conducted examining the prevalence and factors associated with men’s sexual attitudes, sexual interest in, and sexual behaviours towards children. The survey aimed to gather information about the demographic, behavioural and attitudinal correlates of men who are abusing or pose a risk to children to inform the development of coordinated policy and practice responses to child sexual exploitation and abuse. The survey asked a range of questions about men’s backgrounds, childhood experiences, health status, online behaviours, and social context with the intention of identifying opportunities to more accurately target prevention interventions, and to gain a holistic perspective on the pathways towards offending and associated risk factors.

Data were drawn from three census-matched samples of men aged 18 years or over, representative of the Australian, U.K., and U.S. male populations in terms of age, residential region, annual household income, and educational attainment. Survey recruitment and administration were conducted by CloudResearch (https://www.cloudresearch.com), an online research panel company with access to an international pool of over 100 million participants. The survey was reviewed by a project advisory group that included representatives from law enforcement, financial intelligence units, government departments, and mental health support services. Westpac Bank provided funding from its ‘Safer Children, Safer Communities’ programme. As such, some of the survey questions were informed by the broader aims of the study’s funding source, which is concerned with understanding how individuals interact with online services, including financial platforms.

Surveys were administered from November to December 2022. Ethical approval for this study was provided by the University of New South Wales (HC220317).

Initial data were provided by 7,334 respondents (Australia = 2,697; United Kingdom = 2,240; United States = 2,240). Participants were informed that their responses would be entirely anonymous as part of the consent process. However, in line with mandatory reporting requirements, participants were advised not to provide identifying information, as any such details indicating a child was at risk would necessitate a report to relevant authorities. Participants were excluded if they indicated that they were either female at birth, did not identify as male, failed the mid-survey attention check, or reported that they had not answered the questions honestly (*n* = 2,348 removed). Selection bias was reduced by applying population weights to the Australian (*n* = 1,939), United States (*n* = 1,473), and United Kingdom (*n* = 1,506) samples using iterative proportional fitting based on six demographic factors (i.e. race, marital status, employment status, age, annual household income, and educational attainment) sourced from each country’s respective 2021 census. For the current study, data from the three countries were pooled (*n* = 4,918) to increase statistical power.

### Measures

#### Sexual Interest in Children or Sexual Behaviour Involving Minors

Participants were asked 10 questions regarding whether, during adulthood, they had any sexual interest toward children or engaged in sexual behaviour involving minors. Participants were coded as having sexual interest in children if they answered yes to any of the following questions: (a) I would have sexual contact with a child between 12 and 14 years if no one would find out; (b) I would have sexual contact with a child between 10 and 12 years if no one would find out; (c) I would have sexual contact with a child younger than 10 years if no one would find out; (d) the lowest age I typically find attractive is 15 years or younger, and; (e) the highest age I typically find attractive is 15 years or younger.

These questions were taken from two previous studies, ‘Interest in Sex with Children’ (Seto et al., 2015) and ‘Age of Attraction’ (Ó Ciardha et al., 2022). The measure from Seto et al. (2015) is designed to assess hypothetical sexual interest in children. The age-of-attraction questions were adapted from Ó Ciardha et al. (2022). Participants were first prompted with the instruction: ‘Think about the people you typically find most sexually attractive’. They were then asked to report the lowest and highest age they typically find attractive. The upper age threshold of 15 was selected to align with age-of-consent laws in Australia, the United Kingdom, and the United States. This approach captures self-reported attraction patterns relevant to legal and clinical frameworks.

Participants were coded as having engaged in sexual behaviour involving minors if they answered yes to any of the following five questions: (a) I knowingly and deliberately view pornographic material containing people below the age of 18; (b) I have flirted or had sexual conversations with a person below the age of 18 online; (c) I have engaged in a sexually explicit webcam interaction with a person below the age of 18; (d) I have paid for online sexual interactions, images or videos involving a person below the age of 18; (e) and I have had sex or sexual contact with a person below the age of 18. Four non-overlapping categories were created, indicating if participants had reported (a) no sexual interest or behaviour involving minors, (b) sexual interest without behaviour, (c) sexual behaviour without interest, and (d) sexual interest and behaviour.

Children in this study are defined as individuals aged 15 or younger, consistent with the phrasing used in the survey items. Minors in our study were defined as individuals under 18 years old, consistent with international child protection standards (UNICEF, 1989). Accessing, transmitting and soliciting online sexual material of someone under the age of 18 years is also illegal in all countries covered in this study. However, we acknowledge that age-of-consent laws vary across jurisdictions. As such, some self-reported behaviours involving minors may reflect age-proximate, consensual relationships that are not legally classified as offending in all contexts. To address this limitation, we conducted additional subgroup analyses to examine whether participants reporting offline sexual contact with individuals under 18 also demonstrated other risk factors or correlates of child sexual offending. These analyses suggested that while some participants may have reported consensual age relationships, the majority of those reporting sexual contact with individuals under 18 also reported behaviours or characteristics associated with a higher risk for child sexual offending (see Whitten et al., 2026).

##### Demographic Characteristics

Ten demographic factors were included in this study. These were age (1 = 18–24 years; 2 = 25–34 years; 3 = 35–44 years; 4 = 45–54 years; 5 = 55–64 years; 6 = 65 years and older), total U.S. standardised household income before taxes during the last 12 months (1 = less than US$25,000 equivalent; 2 = between US$25,000–US$99,999; 3 = US$100,000 or more), residential location (1 = city; 2 = suburb; 3 = rural or regional), sexual orientation (0 = not heterosexual; 1 = heterosexual), ever had sex with men (0 = no; 1 = yes), relationship status (0 = single, widowed, divorced, or separated; 1 = married or living with partner), educational attainment (0 = did not obtain a bachelor’s degree; 1 = bachelor’s degree or higher), employment status in the last 3 months (0 = unemployed; 1 = casual, part-time, or full-time employment), number of children living in household (0 = none; 1 = one or more), and current occupation involves contact with children (0 = no; 1 = yes).

##### Online Behaviours

Participants indicated the frequency (1 = never, 5 = daily) of their engagement in 13 online behaviours: (a) using search engines; (b) sending emails; (c) using social media; (d) engaging in online blogs; (e) shopping online; (f) online banking; (g) online messaging; (h) private video chatting; (i) livestreaming self; (j) streaming movies; (k) using romance websites or dating apps; (l) online gaming; and (m) watching online pornography. Participants also indicated if, during adulthood, they knowingly and deliberately viewed pornography involving sex between humans and animals (0 = no; 1 = yes), and sex involving violence or force (0 = no; 1 = yes). Finally, participants indicated whether they used any services or software to prevent their online activities from being tracked and surveilled, such as the onion router or virtual private network (0 = no; 1 = yes).

##### Mental Health

Symptoms of anxiety and depression were measured using four items from the Patient Health Questionnaire 4 (PHQ-4) (Löwe et al., 2010). The PHQ-4 asks respondents how often (1 = not at all; 2 = 1–7 days; 3 = 8–11 days; 4 = 12–14 days) over the past 2 weeks had they been bothered by: (a) little interest or pleasure in doing things; (b) feeling down, depressed, or hopeless; (c) feeling nervous, anxious, or on edge; and (d) not being able to stop or control worrying. Scores were summed so that higher scores reflect greater frequency of anxiety and depression symptoms (α = .89). Substance use frequency was assessed using four questions from the National Institute on Drug Abuse (NIDA) Quick Screen v1.0 (https://www.drugabuse.gov/nmassist/). Participants indicated how often (1 = never; 5 = daily or almost daily) over the past year they: (a) drank five or more alcoholic drinks a day (i.e. binge drink); (b) used tobacco products; (c) used prescription drugs for non-medical reasons; and (d) used illicit drugs.

##### Social Support

The availability of social supports was measured using the Multidimensional Scale of Perceived Social Support (MSPSS; Zimet et al., 1990). The MSPSS includes 12 questions responded to on a 7-point Likert scale (1 = *very strongly disagree*; 7 = *very strongly agree*) that correspond to social support from significant others (α = .94), family (α = .92), and friends (α = .93).

##### Adverse Childhood Experiences

Participants reported if, at any time prior to the age of 18 years, they experienced emotional abuse, physical abuse, sexual abuse, low family support, neglect, parental divorce, domestic violence, household drug abuse, household mental illness, or household member incarcerated (Felitti et al., 1998).

##### Attitudes Towards Child Sexual Abuse

Twenty-five items adapted from the Child Sexual Abuse Myth Scale were used to measure men’s attitudes towards online child sexual exploitation (Salter et al., 2026). The scores for each item ranged from ‘*strongly disagree*’ (1) to ‘*strongly agree*’ (5). Principal components analysis was conducted to reduce the 25 items into multiple subscales reflecting different underlying dimensions. Four components were identified, one of which only comprised of two items (α = .73) and was not retained. The three remaining components were designated ‘denial of abusiveness’ (14 items, α = .92), ‘blame diffusion’ (5 items, α = .87), and ‘restrictive stereotypes’ (4 items, α = .81). The average score for each subscale was calculated, with higher scores indicating greater endorsement of attitudes.

### Analytical Strategy

First presented are the descriptive statistics regarding unique categories of men designated as those with (a) no sexual interest or behaviour involving minors, (b) sexual interest without behaviour, (c) sexual behaviour without interest, and (d) sexual interest and behaviour. Next, unadjusted Odds ratios (OR) were computed to identify the variables associated with each combination of the outcome categories. A series of LASSO logistic regression analyses were then conducted to identify the most parsimonious combination of covariates that were independently associated with men who have sexual interest without behaviour (model 1), sexual behaviour without interest (model 2), and sexual interest and behaviour (model 3), relative to the no sexual interest or behaviour group. Additional comparisons were conducted between the sexual behaivour without interest versus sexual interest without behaviour groups (model 4), sexual interest and behaviour versus sexual interest without behaviour groups (model 5), and sexual behaviour and interest versus sexual behaviour without interest groups (model 6). Items identified via this variable selection process were then included in separate logistic regression models to calculate their non-penalised coefficients (expressed as ORs).

LASSO is a regularised method for variable selection and shrinkage, and generally produces greater predictive accuracy than stepwise or best subset variable selection (Hastie et al., 2020). Regularisation introduces a penalty proportional to the absolute values of the regression coefficients, which prevents overfitting and simplifies the model by shrinking some coefficients to zero, effectively excluding irrelevant variables (Tibshirani, 1996). K-fold cross-validation with 10 folds was also conducted to improve the robustness and generalisability of the variable selection process while optimising the regularisation parameter (λ) (Jung, 2018). This divided the dataset into 10 subsets (folds), with the model trained on 9 folds while the 10th is used for validation. This process is repeated across all folds, and the λ that minimises prediction error is selected.

Analyses were conducting using a complex sample design, with standard errors adjusted to account for poststratification weights (Lumley, 2004). Model fit was assessed using deviance (standard error) and Nagelkerke R2. Discrimination accuracy was determined using the area under the receiver operating characteristic curve (AUROC). The model’s capability to distinguish between the comparison and outcome categories was considered poor if AUROC scores ranged from 0.6 to 0.7, fair if scores ranged from 0.7 to 0.8, good if scores ranged from 0.8 to 0.9, and excellent if scores were greater than 0.9 (Nahm, 2022). ORs and the 99% confidence intervals (99% CIs) are reported as measures of effect size and precision of the association between the covariates and outcome variables. All assumptions underpinning logistic regression analysis were met (Tabachnick & Fidell, 2013). Analyses were conducted using IBM SPSS 29 (IBM Corp, 2022) and R version 4.4.2, with the svyglm (Lumley et al., 2024) and glmnet packages (Friedman et al., 2023).

## Results

Most participants reported no sexual interest or behaviour involving minors (83.5% [99% CI = 81.9, 85.1]), around 1-in-18 reported sexual interest without behaviour (5.5% [4.5, 6.6]), 1-in-15 reported sexual behaviour without interest (6.4% [5.4, 7.5]), and almost 1-in-20 reported both sexual interest and behaviour (4.6% [3.8, 5.6]). Table 1 presents the descriptive statistics relating to demographic characteristics, online behaviours, mental health, social support, adverse childhood experiences, and attitudes toward child sexual abuse, separately for each category of sexual interest and behaviour involving minors.

**Table 1.** Descriptive Statistics and Standard Errors of Men Who Have (a) *No Sexual Interest or Behaviour Towards Children*, (b) *Sexual Interest Without Behaviour*, (c) *Sexual Behaviour Without Interest*, and (d) *Sexual Interest and Behaviour*.

Demographic characteristics	No interest or behaviour (*n* = 4,109)	Interest only (*n* = 269)	Behaviour only (*n* = 313)	Interest and behaviour (*n* = 227)
Heterosexual	92.8% (0.5%)	92.8% (1.9%)	92.7% (1.6%)	95.1% (1.8%)
Had sex with men	13.7% (0.6%)	33.1% (3.5%)	19.6% (2.8%)	41.4% (3.7%)
Married or living with partner	59.6% (0.9%)	52.6% (3.7%)	58.6% (3.3%)	77.0% (3.3%)
Employed	64.6% (0.9%)	70.7% (3.5%)	60.7% (3.3%)	86.1% (2.9%)
Bachelor’s degree or higher	36.4% (0.9%)	37.3% (3.5%)	39.7% (3.3%)	51.0% (3.7%)
Child in household	29.8% (0.8%)	41.1% (3.6%)	37.3% (3.3%)	66.5% (3.5%)
Works with children	14.1% (0.6%)	14.6% (2.5%)	18.5% (2.6%)	51.1% (3.7%)
Age				
18–24 years	12.4% (0.6%)	17.8% (2.9%)	14.6% (2.4%)	15.1% (3.0%)
25–34 years	16.2% (0.6%)	33.1% (3.5%)	12.5% (2.1%)	32.3% (3.5%)
35–44 years	16.3% (0.7%)	19.8% (2.7%)	16.9% (2.4%)	26.5% (3.3%)
45–54 years	17.2% (0.7%)	8.1% (2.0%)	13.1% (2.4%)	11.0% (2.3%)
55–64 years	16.6% (0.7%)	9.7% (2.3%)	16.1% (2.5%)	4.0% (1.5%)
65 years and older	21.4% (0.8%)	11.5% (2.5%)	26.8% (2.9%)	11.2% (1.7%)
Household income				
Less than US$25,000	22.0% (0.8%)	29.6% (3.6%)	25.1% (3.0%)	16.7% (3.1%)
US$25,000–US$99,999	56.2% (0.9%)	40.1% (3.6%)	49.2% (3.3%)	43.5% (3.7%)
US$100,000 or more	21.8% (0.8%)	30.3% (3.3%)	25.7% (3.0%)	39.7% (3.6%)
Residential location				
City	29.9% (0.8%)	53.7% (3.7%)	38.0% (3.2%)	74.9% (3.3%)
Suburbs	45.7% (0.9%)	35.8% (3.6%)	42.0% (3.3%)	17.0% (3.0%)
Regional or rural	24.4% (0.8%)	10.6% (2.1%)	19.9% (2.7%)	8.1% (1.9%)
Country				
Australia	39.7% (0.9%)	46.7% (3.7%)	38.3% (3.3%)	27.6% (3.3%)
United Kingdom	31.6% (0.8%)	19.8% (3.0%)	26.5% (2.9%)	30.6% (3.5%)
United States	28.7% (0.8%)	33.6% (3.4%)	35.2% (3.1%)	41.8% (3.7%)
Online behaviours				
Online browsing frequency	4.71 (0.01)	4.32 (0.09)	4.63 (0.05)	4.47 (0.06)
Emailing frequency	4.29 (0.02)	3.89 (0.11)	4.16 (0.07)	4.11 (0.10)
Social media use frequency	4.21 (0.03)	4.19 (0.09)	4.06 (0.10)	4.29 (0.08)
Online blogs frequency	2.83 (0.03)	3.22 (0.12)	2.94 (0.10)	4.06 (0.09)
Online shopping frequency	2.90 (0.02)	3.42 (0.10)	3.08 (0.08)	3.77 (0.08)
Online banking frequency	3.94 (0.02)	3.79 (0.09)	3.97 (0.07)	4.04 (0.08)
Online messaging frequency	3.76 (0.03)	3.70 (0.11)	3.81 (0.10)	4.24 (0.07)
Private video chatting frequency	2.73 (0.03)	3.32 (0.11)	2.84 (0.11)	3.84 (0.09)
Livestream self frequency	2.16 (0.03)	3.28 (0.12)	2.53 (0.11)	3.78 (0.10)
Streaming videos frequency	3.66 (0.03)	3.72 (0.11)	3.60 (0.10)	4.07 (0.08)
Romance/dating sites frequency	1.81 (0.03)	2.77 (0.12)	2.23 (0.10)	3.68 (0.10)
Online gaming frequency	2.43 (0.03)	3.02 (0.11)	2.59 (0.10)	3.53 (0.09)
Online pornography frequency	2.44 (0.03)	2.80 (0.12)	2.81 (0.10)	3.72 (0.09)
Watches violent or rough porn	8.6% (0.5%)	8.0% (1.9%)	21.9% (2.7%)	44.9% (3.7%)
Watches bestiality	2.7% (0.3%)	5.7% (2.0%)	16.6% (2.5%)	33.3% (3.5%)
Use privacy software	46.8% (0.9%)	65.3% (3.6%)	55.8% (3.3%)	91.6% (2.0%)
Mental health				
Binge drinking frequency	2.27 (0.02)	2.45 (0.10)	2.45 (0.09)	3.01 (0.09)
Smoking frequency	2.24 (0.03)	2.46 (0.11)	2.32 (0.11)	3.15 (0.10)
Prescription drug misuse frequency	1.51 (0.02)	2.11 (0.09)	1.85 (0.09)	2.75 (0.10)
Illegal drug use frequency	1.39 (0.02)	1.82 (0.09)	1.60 (0.08)	2.65 (0.11)
Anxiety and depression (PHQ-4)	2.57 (0.05)	3.26 (0.21)	2.64 (0.19)	5.37 (0.23)
Social support				
Support from significant others	5.09 (0.03)	4.38 (0.14)	5.03 (0.10)	5.13 (0.10)
Support from family	4.88 (0.03)	4.41 (0.14)	4.92 (0.10)	5.08 (0.10)
Support from friends	4.68 (0.03)	4.46 (0.13)	4.84 (0.10)	5.13 (0.10)
Adverse childhood experiences				
Childhood emotional abuse	25.5% (0.8%)	26.8% (3.3%)	34.7% (3.2%)	59.4% (3.7%)
Childhood physical abuse	21.1% (0.8%)	25.8% (3.2%)	27.1% (2.9%)	53.9% (3.7%)
Childhood sexual abuse	9.0% (0.5%)	18.1% (2.8%)	18.5% (2.5%)	50.9% (3.7%)
Childhood with low family support	20.9% (0.7%)	24.7% (3.2%)	24.6% (2.8%)	48.3% (3.7%)
Childhood neglect	8.6% (0.5%)	18.0% (2.8%)	15.2% (2.3%)	44.1% (3.7%)
Childhood parental divorce	29.8% (0.8%)	27.6% (3.5%)	30.2% (3.0%)	38.7% (3.6%)
Childhood domestic violence	9.4% (0.5%)	14.8% (2.7%)	15.5% (2.4%)	37.9% (3.6%)
Childhood family drug use	17.9% (0.7%)	21.6% (3.0%)	22.4% (2.7%)	41.2% (3.7%)
Childhood family mental illness	15.1% (0.7%)	13.9% (2.3%)	22.9% (2.8%)	37.4% (3.6%)
Childhood family incarceration	7.4% (0.5%)	15.5% (2.8%)	15.1% (2.4%)	32.2% (3.5%)
Attitudes towards child sex abuse				
Denial of abusiveness	1.87 (0.01)	2.54 (0.07)	2.27 (0.05)	3.41 (0.06)
Blame diffusion	1.94 (0.02)	2.97 (0.08)	2.20 (0.05)	2.49 (0.06)
Restrictive stereotypes	2.34 (0.02)	2.69 (0.08)	2.63 (0.06)	3.47 (0.07)

*Note*. PHQ-4 = Patient Health Questionnaire 4.

Table 2 presents the unadjusted associations (ORs) between the covariates and each combination of the outcome categories. Sexual orientation, social media use, and online banking were the only variables not significantly associated with any outcome category. By contrast, all outcome categories differed significantly from one another in terms of the frequency of online shopping (ORs = 0.78 [99% CI = 0.61, 0.88] to 1.71 [99% CI = 1.35, 2.16]), livestreaming oneself (0.77 [0.66, 0.89] to 1.59 [1.37, 1.86]), and engagement with romance websites or dating apps (0.81 [0.70, 0.95] to 1.74 [1.49, 2.04]), as well as attitudes related to denial of abusiveness (0.67 [0.48, 0.93] to 10.04 [7.43, 13.58]) and blame diffusion (0.55 [0.45, 0.67] to 2.46 [2.08, 2.91]). Most variables were significantly associated with the sexual interest and behaviour group, with the greatest effect sizes for watches bestiality (18.27 [11.05, 30.23]), uses privacy software (12.44 [6.21, 24.90]), and childhood sexual abuse (10.56 [6.95, 16.04]), relative to those with no sexual interest or behaviour. Conversely, the fewest significant differences overall were for the sexual behaviour without interest group.

**Table 2.** Unadjusted odds [99% CI].

	No interest or behaviour (reference group)			Interest only (reference group)		Behaviour only (reference group)
	Interest only	Behaviour only	Interest and behaviour	Behaviour only	Interest and behaviour	Interest and behaviour
Demographic characteristics	OR [99% CI]	OR [99% CI]	OR [99% CI]	OR [99% CI]	OR [99% CI]	OR [99% CI]
Heterosexual	1.00 [0.47, 2.13]	0.99 [0.53, 1.86]	1.49 [0.55, 4.06]	0.99 [0.38, 2.55]	1.49 [0.44, 5.07]	1.51 [0.47, 4.80]
Had sex with men	3.12 [2.04, 4.78]	1.54 [0.96, 2.47]	4.46 [2.95, 6.73]	0.49 [0.27, 0.91]	1.43 [0.82, 2.50]	2.89 [1.59, 5.25]
Married or living with partner	0.75 [0.51, 1.12]	0.96 [0.67, 1.38]	2.27 [1.39, 3.70]	1.28 [0.76, 2.15]	3.03 [1.64, 5.59]	2.37 [1.31, 4.30]
Employed	1.33 [0.85, 2.08]	0.85 [0.59, 1.22]	3.41 [1.80, 6.47]	0.64 [0.36, 1.12]	2.57 [1.19, 5.55]	4.02 [1.95, 8.29]
Bachelor’s degree or higher	1.04 [0.70, 1.55]	1.15 [0.80, 1.66]	1.82 [1.23, 2.70]	1.11 [0.66, 1.87]	1.75 [1.01, 3.02]	1.58 [0.94, 2.66]
Child in household	1.64 [1.10, 2.44]	1.40 [0.97, 2.04]	4.67 [3.06, 7.11]	0.86 [0.51, 1.44]	2.84 [1.63, 4.98]	3.33 [1.93, 5.73]
Works with children	1.04 [0.61, 1.77]	1.39 [0.87, 2.20]	6.38 [4.26, 9.56]	1.33 [0.67, 2.64]	6.13 [3.22, 11.67]	4.60 [2.56, 8.28]
Age	0.75 [0.66, 0.85]	1.04 [0.93, 1.16]	0.74 [0.67, 0.82]	1.38 [1.17, 1.62]	0.99 [0.84, 1.15]	0.72 [0.62, 0.83]
Household income						
Less than US$25,000	1.88 [1.15, 3.10]	1.30 [0.83, 2.05]	0.98 [0.53, 1.83]	0.69 [0.36, 1.32]	0.52 [0.24, 1.13]	0.75 [0.36, 1.60]
US$25,000–US$99,999	1.00 [reference]	1.00 [reference]	1.00 [reference]	1.00 [reference]	1.00 [reference]	1.00 [reference]
US$100,000 or more	1.96 [1.25, 3.08]	1.35 [0.88, 2.08]	2.36 [1.55, 3.59]	0.69 [0.38, 1.26]	1.21 [0.66, 2.19]	1.75 [0.98, 3.12]
Residential location						
City	2.29 [1.49, 3.54]	1.38 [0.93, 2.06]	6.73 [3.82, 11.83]	0.60 [0.34, 1.06]	2.93 [1.47, 5.87]	4.86 [2.48, 9.53]
Suburbs	1.00 [reference]	1.00 [reference]	1.00 [reference]	1.00 [reference]	1.00 [reference]	1.00 [reference]
Regional or rural	0.55 [0.29, 1.06]	0.89 [0.55, 1.44]	0.90 [0.39, 2.05]	1.61 [0.73, 3.54]	1.62 [0.57, 4.56]	1.01 [0.39, 2.58]
Online behaviours						
Online browsing frequency	0.60 [0.48, 0.74]	0.86 [0.68, 1.10]	0.69 [0.57, 0.84]	1.45 [1.07, 1.96]	1.17 [0.90, 1.51]	0.81 [0.61, 1.07]
Emailing frequency	0.75 [0.63, 0.89]	0.90 [0.78, 1.05]	0.87 [0.72, 1.04]	1.21 [0.97, 1.50]	1.16 [0.91, 1.48]	0.96 [0.76, 1.21]
Social media use frequency	0.99 [0.87, 1.13]	0.93 [0.83, 1.05]	1.05 [0.92, 1.19]	0.94 [0.79, 1.12]	1.06 [0.89, 1.26]	1.12 [0.95, 1.33]
Online blogs frequency	1.16 [1.03, 1.32]	1.04 [0.94, 1.17]	1.75 [1.50, 2.04]	0.90 [0.77, 1.05]	1.50 [1.24, 1.82]	1.68 [1.40, 2.01]
Online shopping frequency	1.46 [1.19, 1.78]	1.15 [1.00, 1.32]	1.94 [1.60, 2.35]	0.78 [0.61, 0.99]	1.33 [1.02, 1.74]	1.71 [1.35, 2.16]
Online banking frequency	0.89 [0.76, 1.05]	1.02 [0.88, 1.20]	1.08 [0.91, 1.30]	1.15 [0.93, 1.43]	1.22 [0.96, 1.54]	1.06 [0.84, 1.34]
Online messaging frequency	0.97 [0.86, 1.10]	1.02 [0.91, 1.16]	1.29 [1.14, 1.46]	1.05 [0.89, 1.24]	1.33 [1.12, 1.57]	1.26 [1.07, 1.49]
Private video chatting frequency	1.30 [1.14, 1.48]	1.05 [0.93, 1.19]	1.71 [1.49, 1.97]	0.81 [0.68, 0.96]	1.32 [1.09, 1.58]	1.63 [1.36, 1.95]
Livestream self frequency	1.51 [1.34, 1.71]	1.16 [1.04, 1.29]	1.84 [1.64, 2.07]	0.77 [0.66, 0.89]	1.22 [1.04, 1.43]	1.59 [1.37, 1.86]
Streaming videos frequency	1.02 [0.90, 1.16]	0.97 [0.87, 1.09]	1.23 [1.09, 1.38]	0.95 [0.81, 1.12]	1.20 [1.01, 1.42]	1.26 [1.08, 1.48]
Romance/dating sites frequency	1.50 [1.34, 1.69]	1.22 [1.09, 1.37]	2.13 [1.88, 2.40]	0.81 [0.70, 0.95]	1.42 [1.21, 1.66]	1.74 [1.49, 2.04]
Online gaming frequency	1.26 [1.12, 1.41]	1.07 [0.96, 1.19]	1.54 [1.38, 1.72]	0.85 [0.73, 0.99]	1.23 [1.05, 1.43]	1.44 [1.25, 1.68]
Online pornography frequency	1.17 [1.02, 1.35]	1.18 [1.05, 1.33]	1.83 [1.59, 2.10]	1.00 [0.84, 1.20]	1.56 [1.28, 1.89]	1.55 [1.30, 1.85]
Watches violent or rough porn	0.93 [0.46, 1.86]	2.98 [1.92, 4.61]	8.62 [5.63, 13.20]	3.21 [1.47, 7.03]	9.31 [4.29, 20.23]	2.90 [1.66, 5.05]
Watches bestiality	2.21 [0.81, 6.02]	7.29 [4.22, 12.58]	18.27 [11.05, 30.23]	3.30 [1.14, 9.55]	8.28 [2.92, 23.45]	2.51 [1.36, 4.63]
Use privacy software	2.14 [1.40, 3.26]	1.44 [1.01, 2.05]	12.44 [6.21, 24.90]	0.67 [0.39, 1.15]	5.81 [2.61, 12.95]	8.66 [4.01, 18.68]
Mental health						
Binge drinking frequency	1.10 [0.96, 1.27]	1.10 [0.97, 1.25]	1.47 [1.30, 1.66]	1.00 [0.84, 1.20]	1.33 [1.11, 1.58]	1.33 [1.13, 1.57]
Smoking frequency	1.08 [0.98, 1.20]	1.03 [0.93, 1.15]	1.35 [1.24, 1.47]	0.96 [0.83, 1.10]	1.25 [1.10, 1.42]	1.31 [1.15, 1.49]
Prescription drug misuse frequency	1.47 [1.30, 1.66]	1.28 [1.11, 1.47]	1.90 [1.70, 2.12]	0.87 [0.74, 1.04]	1.29 [1.12, 1.50]	1.48 [1.26, 1.74]
Illegal drug use frequency	1.39 [1.21, 1.60]	1.21 [1.03, 1.43]	1.99 [1.76, 2.24]	0.87 [0.71, 1.07]	1.43 [1.21, 1.68]	1.64 [1.36, 1.98]
Anxiety and depression (PHQ-4)	1.08 [1.01, 1.14]	1.01 [0.95, 1.07]	1.28 [1.22, 1.34]	0.94 [0.86, 1.01]	1.18 [1.11, 1.27]	1.27 [1.18, 1.36]
Social support						
Support from significant others	0.79 [0.71, 0.88]	0.98 [0.88, 1.08]	1.01 [0.91, 1.12]	1.24 [1.07, 1.43]	1.28 [1.11, 1.48]	1.04 [0.90, 1.19]
Support from family	0.84 [0.74, 0.95]	1.02 [0.91, 1.15]	1.09 [0.97, 1.24]	1.22 [1.03, 1.44]	1.31 [1.10, 1.55]	1.07 [0.91, 1.26]
Support from friends	0.91 [0.79, 1.05]	1.07 [0.95, 1.21]	1.24 [1.08, 1.44]	1.18 [0.98, 1.41]	1.36 [1.12, 1.67]	1.16 [0.96, 1.40]
Adverse childhood experiences						
Childhood emotional abuse	1.07 [0.69, 1.66]	1.51 [1.06, 2.26]	4.26 [2.84, 6.40]	1.45 [0.83, 2.54]	3.99 [2.24, 7.12]	2.75 [1.62, 4.68]
Childhood physical abuse	1.31 [0.83, 2.05]	1.39 [0.93, 2.08]	4.39 [2.94, 6.55]	1.07 [0.60, 1.90]	3.36 [1.89, 6.00]	3.16 [1.84, 5.42]
Childhood sexual abuse	2.24 [1.34, 3.75]	2.31 [1.45, 3.65]	10.56 [6.95, 16.04]	1.03 [0.54, 1.97]	4.71 [2.54, 8.75]	4.58 [2.58, 8.14]
Childhood with low family support	1.24 [0.79, 1.95]	1.23 [0.82, 1.84]	3.53 [2.37, 5.26]	0.99 [0.55, 1.78]	2.85 [1.59, 5.08]	2.87 [1.67, 4.93]
Childhood neglect	2.33 [1.40, 3.89]	1.91 [1.17, 3.11]	8.36 [5.50, 12.73]	0.82 [0.42, 1.59]	3.58 [1.93, 6.64]	4.38 [2.41, 7.98]
Childhood parental divorce	0.90 [0.58, 1.40]	1.02 [0.70, 1.49]	1.49 [1.00, 2.23]	1.13 [0.64, 2.00]	1.66 [0.92, 2.97]	1.46 [0.86, 2.50]
Childhood domestic violence	1.68 [0.95, 2.98]	1.76 [1.07, 2.91]	5.89 [3.84, 9.05]	1.05 [0.51, 2.17]	3.51 [1.78, 6.92]	3.34 [1.80, 6.21]
Childhood family drug use	1.26 [0.78, 2.03]	1.32 [0.87, 2.02]	3.21 [2.13, 4.84]	1.05 [0.57, 1.94]	2.55 [1.40, 4.67]	2.43 [1.38, 4.26]
Childhood family mental illness	0.91 [0.54, 1.53]	1.68 [1.09, 2.57]	3.37 [2.23, 5.10]	1.84 [0.97, 3.52]	3.70 [1.96, 7.01]	2.01 [1.14, 3.53]
Childhood family incarceration	2.30 [1.30, 4.07]	2.23 [1.33, 3.74]	5.96 [3.81, 9.31]	0.97 [0.47, 2.01]	2.59 [1.31, 5.10]	2.67 [1.42, 5.04]
Attitudes towards child sex abuse						
Denial of abusiveness	3.21 [2.45, 4.21]	2.14 [1.70, 2.70]	10.04 [7.43, 13.58]	0.67 [0.48, 0.93]	3.13 [2.18, 4.49]	4.69 [3.33, 6.60]
Blame diffusion	2.46 [2.08, 2.91]	1.34 [1.16, 1.56]	1.74 [1.50, 2.03]	0.55 [0.45, 0.67]	0.71 [0.58, 0.86]	1.30 [1.08, 1.57]
Restrictive stereotypes	1.64 [1.23, 2.18]	1.51 [1.21, 1.88]	4.90 [3.67, 6.56]	0.92 [0.65, 1.31]	2.99 [2.01, 4.44]	3.25 [2.29, 4.60]

Table 3 presents the results of the logistic regression models developed via LASSO variable selection comparing categories of sexual interest and behaviour involving minors. Models 1 to 3 compare men reporting sexual interest without behaviour (model 1), sexual behaviour without interest (model 2), and sexual interest and behaviour (model 3), relative to the no sexual interest or behaviour group. In model 1, the sexual interest without behaviour group were significantly more likely to livestream themself (OR = 1.25 [99% CI = 1.05, 1.50]) and endorse denial of abusiveness (2.64 [1.79, 3.89]) and blame diffusion attitudes (2.57 [1.95, 3.40]). This model had a deviance of 0.34, good discriminant accuracy (AUROC = 0.87) and explained 32% of the variance. Model 2 indicated that the sexual behaviour without interest group was significantly more likely to watch bestiality (5.01 [2.84, 8.82]) and endorse denial of abusiveness attitudes (1.69 [1.23, 2.32]). This model had a deviance of 0.47, fair discriminant accuracy (AUROC = .71), and explained 12% of the variance. In model 3, the sexual interest and behaviour group were significantly more likely live in the city (2.32 [1.02, 5.32]), watch violent pornography (5.68 [2.45, 13.14]) and bestiality (2.69 [1.05, 6.86]), use privacy software (3.26 [1.20, 8.85]), have higher PHQ-4 scores (1.17 [1.03, 1.34]), a childhood history of sexual abuse (2.38 [1.09, 5.19]), be less likely to have experienced parental divorce (0.38 [0.15, 0.90]), and endorse denial of abusiveness (5.01 [2.86, 8.76]) and blame diffusion attitudes (2.56 [1.74, 3.76]). This model had the lowest deviance (0.18), the highest discriminant accuracy (AUROC = 0.98), and the highest variance explained (69%).

**Table 3.** Logistic Regression With LASSO *k*-Fold (10) Variable Selection (Adjusted for Country; Variables Not Selected Excluded From Table).

	No interest or behaviour (reference group)			Interest only (reference group)		Behaviour only (reference group)
	Model 1	Model 2	Model 3	Model 4	Model 5	Model 6
	Interest only	Behaviour only	Interest and behaviour	Behaviour only	Interest and behaviour	Interest and behaviour
Demographic characteristics	OR [99% CI]	OR [99% CI]	OR [99% CI]	OR [99% CI]	OR [99% CI]	OR [99% CI]
Heterosexual	–	–	4.66 [0.79, 27.68]	–	–	3.83 [0.77, 19.18]
Had sex with men	–	–	1.88 [0.85, 4.16]	0.59 [0.29, 1.22]	–	2.09 [0.78, 5.62]
Married or living with a partner	1.01 [0.61, 1.65]	–	1.79 [0.76, 4.25]	–	2.21 [0.96, 5.11]	2.37 [1.00, 5.64]
Employed	–	–	–	–	–	1.36 [0.43, 4.28]
Bachelor’s degree or higher	–	1.14 [0.74, 1.77]	0.52 [0.24, 1.12]	–	–	0.18 [0.07, 0.47]
Child in household	–	–	1.32 [0.54, 3.25]	–	–	1.52 [0.64, 3.58]
Works with children	0.68 [0.35, 1.34]	1.03 [0.62, 1.74]	1.25 [0.59, 2.65]	1.40 [0.60, 3.29]	2.46 [1.00, 6.12]	–
Age	–	–	1.13 [0.90, 1.40]	1.25 [1.00, 1.58]	–	–
Household income						
Less than US$25,000	1.68 [0.93, 3.02]	1.11 [0.66, 1.85]	–	0.57 [0.25, 1.28]	0.80 [0.25, 2.52]	–
US$25,000–US$99,999	1.00 [reference]	1.00 [reference]	–	1.00 [reference]	1.00 [reference]	–
US$100,000 or more	1.65 [0.98, 2.78]	1.14 [0.70, 1.85]	–	0.82 [0.37, 1.80]	0.94 [0.39, 2.24]	–
Residential location						
City	1.31 [0.79, 2.16]	–	2.32 [1.02, 5.32]	–	1.71 [0.68, 4.26]	2.21 [0.69, 7.01]
Suburbs	1.00 [reference]	–	1.00 [reference]	–	1.00 [reference]	1.00 [reference]
Regional or rural	0.61 [0.29, 1.28]	–	1.08 [0.31, 3.72]	–	2.08 [0.48, 9.02]	3.21 [0.79, 13.08]
Online behaviours						
Online browsing frequency	0.77 [0.57, 1.05]	1.08 [0.82, 1.44]	0.88 [0.58, 1.34]	1.36 [0.93, 1.99]	–	0.98 [0.51, 1.90]
Emailing frequency	0.88 [0.67, 1.14]	0.91 [0.74, 1.12]	–	–	–	–
Social media use frequency	1.08 [0.88, 1.32]	0.97 [0.84, 1.12]	–	0.81 [0.59, 1.11]	–	–
Online blogs frequency	–	–	–	–	1.16 [0.78, 1.70]	1.04 [0.72, 1.51]
Online shopping frequency	–	–	0.79 [0.58, 1.09]	0.91 [0.63, 1.31]	0.63 [0.40, 0.99]	–
Online messaging frequency	–	–	–	1.32 [0.94, 1.86]	1.22 [0.83, 1.79]	–
Private video chatting frequency	–	–	–	0.90 [0.65, 1.24]	–	1.15 [0.78, 1.68]
Livestream self frequency	1.25 [1.05, 1.50]	1.03 [0.89, 1.20]	–	0.87 [0.67, 1.14]	–	–
Romance/dating sites frequency	–	–	1.32 [0.98– 1.77]	–	1.07 [0.76, 1.52]	1.31 [0.96, 1.79]
Online gaming frequency	0.96 [0.80, 1.15]	0.94 [0.82, 1.08]	0.83 [0.61, 1.12]	–	–	–
Online pornography frequency	–	–	1.18 [0.88, 1.58]	1.18 [0.90, 1.56]	1.06 [0.77, 1.45]	–
Watches violent or rough porn	–	–	5.68 [2.45, 13.14]	2.52 [0.89, 7.13]	6.20 [1.89, 20.35]	1.87 [0.82, 4.27]
Watches bestiality	–	5.01 [2.84, 8.82]	2.69 [1.05, 6.86]	3.08 [0.85, 11.14]	2.45 [0.73, 8.24]	–
Use privacy software	–	1.15 [0.76, 1.75]	3.26 [1.20, 8.85]	–	2.46 [0.79, 7.60]	2.02 [0.68, 5.99]
Mental health						
Binge drinking frequency	–	1.01 [0.88, 1.16]	–	–	–	–
Prescription drug misuse frequency	–	1.14 [0.97, 1.34]	1.02 [0.79, 1.33]	–	–	–
Illegal drug use frequency	1.06 [0.88, 1.27]	–	1.01 [0.77, 1.32]	–	–	–
Anxiety and depression (PHQ-4)	–	–	1.17 [1.03, 1.34]	–	1.12 [0.98, 1.29]	1.17 [1.02, 1.33]
Social support						
Support from significant others	–	1.05 [0.92, 1.20]	–	–	–	–
Support from family	0.85 [0.67, 1.07]	–	–	–	–	–
Support from friends	1.07 [0.83, 1.37]	–	1.11 [0.85, 1.45]	1.09 [0.87, 1.36]	–	–
Adverse childhood experiences						
Childhood emotional abuse	–	1.68 [0.94, 2.99]]	1.23 [0.49, 3.10]	1.45 [0.66, 3.21]	1.17 [0.40, 3.39]	–
Childhood physical abuse	–	0.87 [0.47, 1.61]	–	–	1.14 [0.38, 3.42]	0.99 [0.44, 2.20]
Childhood sexual abuse	–	–	2.38 [1.09, 5.19]	–	1.13 [0.39, 3.26]	1.29 [0.55, 3.02]
Childhood with low family support	1.31 [0.73, 2.33]	0.94 [0.56, 1.56]	–	–	–	–
Childhood neglect	–	–	1.66 [0.69, 4.00]	–	–	–
Childhood parental divorce	0.86 [0.50, 1.49]	–	0.37 [0.15, 0.90]	–	–	–
Childhood family mental illness	–	–	–	1.66 [0.69, 4.00]	–	–
Childhood family incarceration	–	1.66 [0.92, 3.00]	1.71 [0.70, 4.16]	0.77 [0.30, 2.01]	–	–
Attitudes towards child sex abuse						
Denial of abusiveness	2.64 [1.79, 3.89]	1.69 [1.23, 2.32]	5.01 [2.86, 8.76]	0.54 [0.36, 0.82]	1.76 [1.12, 2.76]	4.24 [2.44, 7.36]
Blame diffusion	2.57 [1.95, 3.40]	1.18 [0.96, 1.45]	2.56 [1.74, 3.76]	0.52 [0.36, 0.74]	–	2.23 [1.38, 3.59]
Restrictive stereotypes	1.25 [0.83, 1.86]	1.13 [0.84, 1.53]	1.31 [0.80, 2.15]	–	–	–
Deviance (*SE*)	0.34 (0.02)	0.47 (0.02)	0.18 (0.02)	1.15 (0.04)	0.93 (0.05)	0.87 (0.06)
Nagelkerke *R*^2^	0.32	0.12	0.69	0.41	0.56	0.63
AU [99% CI]	0.87 [0.83, 0.90]	0.71 [0.66, 0.75]	0.98 [0.96, 0.99]	0.81 [0.76, 0.87]	0.88 [0.83, 0.92]	0.91 [0.87, 0.94]

*Note*. LASSO, Least Absolute Shrinkage and Selection Operator.

Models 4 and 5 compare the sexual behaviour without interest (model 4) and sexual behaviour and interest groups (model 5) to those with sexual interest without behaviour.

Model 4 indicates that the sexual behaviour without interest group was significantly more likely to be older (1.25 [1.00, 1.58]), and less likely to endorse denial of abusiveness (0.54 [0.36, 0.82]) and blame diffusion attitudes (0.52 [0.36, 0.74]). This model had the highest deviance (1.15) despite achieving good discriminant accuracy (AUROC = 0.81) and explaining 41% of the variance. In model 5, the sexual interest and behaviour group were significantly more likely to work with children (2.46 [1.00, 6.12]), watch violent pornography (6.20 [1.89, 20.35]), and endorse denial of abusiveness attitudes (1.76 [1.12, 2.76]), while being less likely to shop online (0.63 [0.40, 0.99]). Model deviance was 0.93, with good discriminant accuracy (AUROC = .88) and explained 56% of the variance.

Model 6 compared those reporting sexual interest and behaviour to the sexual behaviour without interest group. Those reporting sexual interest and behaviour towards children were significantly more likely to be married or living with a partner (2.37 [1.00, 5.64]), have higher PHQ-4 scores (1.17 [1.02, 1.33]), and endorse denial of abusiveness (4.24 [2.44, 7.36]) and blame diffusion attitudes (2.23 [1.38, 3.59]), while also being significantly less likely to have a bachelor’s degree (0.18 [0.07, 0.47]). Model deviance was 0.87, with excellent discriminant accuracy (AUROC = .91) and explaining 63% of the variance.

## Discussion

The findings of this study show that approximately one in six men reported sexual interest in children and/or sexual behaviour involving minors. It is important to interpret this statistic cautiously, as it reflects a combination of self-reported interest and behaviours that may include sexual activity with minors, yet are not illegal in all jurisdictions. However, our findings suggest that these cohorts of men differed significantly from other men in the community.

Compared to men reporting no sexual interest or behaviour involving minors, men who reported sexual interest without behaviour were more likely to be younger, live in urban areas, have lower incomes and have experienced childhood sexual abuse. They misused prescription drugs, endorsed denial of abusiveness attitudes and had higher levels of psychological distress. Previous studies have suggested that non-offending men with sexual interest in children tend to be younger and have higher levels of psychological distress, and some have characterised this group as potential ‘pre-offenders’ (Cohen et al., 2017; Levine & Dandamudi, 2016). While this interpretation underscores the importance of providing accessible prevention and support services, the observed age differences in our findings could reflect cohort effects, such as younger men being more likely to disclose their sexual interests. Longitudinal measures are needed to assess whether younger non-offending men with sexual interest are at heightened risk of later offending.

Compared to non-offending men without sexual interest in children, men who reported sexual behaviour involving minors but no sexual interest were more likely to be older, have a child in their household, consume deviant pornography and have a childhood history of family mental illness and incarceration of a family member. They had higher PHQ-4 scores and endorsed denial of abusiveness attitudes. These men are the least distinguishable from men in the community who reported neither sexual interest in children nor sexual behaviour involving minors, signifying that they are not markedly different from other men in the community. These findings suggest that engagement in sexual behaviour involving minors may arise due to the interactive effects between individual risk factors and other characteristics, such as cumulative stressors and situational opportunities to offend. It is also possible that their sexual abuse of children is part of a broader pattern of criminality, congruent with forensic descriptions of ‘problems pathway’ offenders (Osbourne & Christensen, 2020). However, our study did not include measures of other criminal behaviours.

Men who reported both sexual interest in children and sexual behaviour involving minors were the most homogenous group of the three categories: those with sexual interest, sexual behaviour, or both. Compared to men without sexual interest in children or sexual behaviour involving minors, they were more likely to be married or living with a partner and work with children. They have a history of childhood sexual abuse and neglect, have higher PHQ-4 scores, and endorse denial of abusive attitudes. This group had significantly higher engagement in high-risk online behaviours, including frequent use of privacy software, viewing violent and bestiality pornography and engaging with romance websites and dating apps. These findings are broadly congruent with forensic descriptions of offending paedophiles who exhibit high levels of sexually deviant interests, are married and have children (Osbourne & Christensen, 2020). Nicol et al.’s (2022) model of the ‘long detection lag’ offender is also applicable. This type of offender is superficially pro-social and trusted by family and colleagues, and is thus able to abuse children for a prolonged period without detection.

Several important similarities among these three groups differentiate them from men who reported neither sexual interest in children nor sexual behaviour involving minors. They had elevated rates of adverse childhood experiences, held permissive attitudes and beliefs about the sexual abuse of children, and had higher rates of depression and anxiety than men in the general population. Men with sexual interest, both those who reported sexual behaviour and those who did not, were more likely to report being sexually abused as children. In contrast, men who reported sexual behaviour involving minors without also reporting sexual interest had higher levels of non-sexual ACEs. Men who reported sexual behaviour involving minors, with or without sexual interest, were more likely to report patterns of deviant pornography consumption relating to violent or bestiality content.

These findings point to the role of adverse childhood experiences in pathways of sexual and behavioural dysregulation to child sexual offending, as well as the role of norms, attitudes and cognitive distortions in justifying the abuse of children. However, causality cannot be inferred from the present data. Increased rates of depression and anxiety have been observed in other studies of child sex offenders (Boillat et al., 2017) and have been understood as a dynamic risk factor that may lead to offending as a maladaptive coping strategy (Seto, 2018). It may also be the case that wanting to, or actually, sexually abusing children may increase depression or anxiety, including fear of detection and punishment.

There were important differences among these three groups. Compared to men with sexual interest in children but no sexual behaviour involving minors, men who reported both sexual interest and sexual behaviour were more likely to be married or living with a partner and working professionally with children. They were also more sexually active online, including more likely to watch violent and bestiality content, use privacy software to obscure their online behaviour, and use romance websites and dating apps. They had higher levels of depression and anxiety than men who did not report sexual behaviour involving minors and were more likely to deny that the sexual abuse of children was harmful. The key factors that distinguished men with sexual interest in children who reported sexual behaviour involving minors from those who did not were essentially their lifestyle and behavioural choices. Men who reported both interest and behaviour were more likely to engage in activities that may increase access to children and inhibit detection (i.e. via privacy software). A similar pattern of associated factors also distinguished between men reported sexual behaviour involving minors without sexual interest from those who reported both sexual behaviour and sexual interest in children.

This is the first study to explore differences among men who report sexual interest in children, men who report sexual behaviour involving minors, and those who report both, using representative community samples from multiple countries. The key strength of the current study, in addition to the design, is the analytical approach to examine different classes of men. By applying LASSO logistic regression with k-fold cross-validation, we were able to identify the most relevant factors that differentiate these groups while minimising overfitting. This approach enhances the nuance of findings across different groups of men, which is critical for developing targeted prevention and response strategies. However, several limitations should be discussed. This study is based on self-reports of sexual interests in children and sexual behaviour involving minors, both of which may be underreported by men due to social desirability and/or recall bias. The finding that one in six men has sexual interest and/or sexual behaviour involving minors should be interpreted in light of the study’s use of a broad definition, in which a minor is defined as an individual under the age of 18. While this definition aligns with international and legal frameworks, it may also capture some consensual relationships that do not constitute illegal behaviour in some jurisdictions. The study also focused on men only, given that they are more likely to sexually offend against children than women. Future research may explore these same types of behaviours and interests among females.

## Conclusion

The findings of this study highlight distinct demographic, behavioural, and health differences among men with sexual interest in children, men without such interest who engage in sexual behaviour involving minors, and men who report both. These findings have several implications for prevention, early intervention, detection and prosecution of offenders. As other studies in this issue have found, adverse childhood experiences, particularly sexual abuse, are risk factors for males later offending against children. Hence, the prevention of ACEs, as well as therapeutic support to abused children, are important prevention strategies. Men who reported sexual interest and/or sexual behaviour involving minors were more likely to hold permissive attitudes towards child sex offending, which suggests that social marketing campaigns emphasising the harms of child sexual abuse and targeting men based on their attitudes to sexual abuse may assist in prevention and early intervention. Men with sexual interest in children who had not engaged in sexual behaviour were younger than those who had, emphasising a prevention window and the need to engage these men in services to manage their risk at a younger age.

The viewing of violent and bestiality pornography among men who reported sexual behaviour involving minors raises questions about the availability and role of deviant pornography in pathways to harmful behaviour. Internet regulation could reduce the availability of this content, while warnings to men who are seeking to access this content (similar to the warnings delivered in some instances to men seeking child sexual abuse material, see Hunn et al., 2023) could refer a broader group of men at risk of harming children to early intervention services. It is important to note that men with men who reported both sexual interest in children and sexual behaviour involving minors, in our study, appeared to have made several personal and professional choices that facilitate their offending and inhibit detection, broadly congruent with other studies of men who sexually abuse children over a long period without coming to the attention of authorities (Nicol et al., 2022). This group often engages in premeditated behaviour, including grooming techniques, and may possess significant social and economic capital to draw on if they are accused of offending. Attempts to thwart this group may include education campaigns for children and institutions about grooming behaviours and removing ‘good character’ evidence in criminal justice and sentencing processes, given that, for this cohort, cultivating a good reputation may be intrinsic to their strategies. This group also preferentially used privacy software, including encrypted social media sites, and hence, proposed legislation that seeks to proactively detect child grooming and child sexual abuse material in encrypted environments could be effective at disrupting the behaviours of this group and reducing harm.
